# Ackonc-AWA: A multi-species animal welfare assessment protocol for wild animals under human care to overcome the use of generic welfare checklists

**DOI:** 10.3389/fvets.2022.1033821

**Published:** 2022-12-08

**Authors:** Débora Silvia Racciatti, Alejandra Feld, Laura Analía Rial, Carlos Blanco, Oriol Tallo-Parra

**Affiliations:** ^1^Cátedra de Bienestar Animal y Etología, Facultad de Ciencias Veterinarias, Universidad de Buenos Aires (UBA), Buenos Aires, Argentina; ^2^Cátedra de Sociología Rural, Facultad de Ciencias Veterinarias, Universidad de Buenos Aires (UBA), Buenos Aires, Argentina; ^3^Zoo Animal Welfare Education Center (ZAWEC), Universitat Autònoma de Barcelona (UAB), Barcelona, Spain; ^4^Departamento de Ciencia Animal y Alimentaria, Facultad de Ciencias Veterinarias, Universitat Autònoma de Barcelona (UAB), Barcelona, Spain

**Keywords:** animal-based measurements, animal welfare, assessment protocol, compassionate conservation, management-based measurements, resource-based measurements, zoo, animal welfare indicators

## Abstract

**Introduction:**

Maintaining a high level of animal welfare is essential in zoos, sanctuaries and aquaria for ethical, legislative and functional reasons. Therefore, it is necessary to have welfare assessment protocols that can be incorporated into daily management programs. Currently, there are different approaches to assessing animal welfare in zoos. Those that can be applied to multiple species consist of checklists or qualitative assessments, with limitations, especially regarding the lack of guidance in the selection and interpretation of indicators. Validated protocols also exist, but they are for very few wild species. This study aimed to develop, test in the field, and describe an animal welfare assessment protocol for wild animals under human care, that can be applied to multiple species, intended to overcome the use of generic welfare checklists and offer an alternative to challenging and time consuming species-specific tools.

**Methods:**

The development process consisted of the elaboration of a protocol, substantiated by published literature on zoo animal welfare and multidisciplinary focus group work, and its on-field feasibility test. This was performed on 14 species of different taxa housed in an Argentinian zoo. The protocol was structured in two forms: an initial form to serve as scan using various animal-based (ABM), resource-based (RBM), and management-based measurements (MBM), and a follow-up form using exclusively ABM. The protocol also included a user's manual with information about preliminary preparation, equipment required, steps from arrival until completion, and details on how to assess each indicator. The scoring method consisted in rating each indicator on a 3-point scale.

**Results:**

23 ABM, 19 RBM, and three MBM were tested and selected to integrate Ackonc-AWA, a multidimensional protocol covering the five animal welfare domains and applicable to multiple species.

**Discussion:**

This protocol was entirely developed in Spanish and can be applied noninvasively and at a low cost, which constitute features of high relevance for Latin America. Further applications of the described welfare assessment tool in other species and different institutional contexts will reinforce the validation of the proposed measurements and allow the systematic and routine evaluation of animal welfare in zoos.

## Introduction

Individual animal welfare and species welfare are critical obligations of zoos, sanctuaries, and aquaria (hereafter simplified as “zoo(s)”). Even the most ambitious conservation goals will not be adequate justification for keeping wild animals in captivity if zoos do not actively demonstrate high standards of animal welfare (1–4). The integration of animal welfare and wildlife conservation has been reflected in the emergence of new fields of study, such as compassionate conservation and conservation welfare. These multidisciplinary approaches attribute intrinsic value to some individual wild animals and support our moral obligation to consider their welfare, interacting with responsibilities to protect other aspects of nature, such as populations, species, ecosystems and biodiversity (5–8). Despite these similarities, there are differences in their ethical foundations, and pragmatism that have been deeply discussed in the literature [e.g., (8, 9)].

The past few decades have seen an increased interest in animal welfare among researchers and zoo staff. Zookeepers identify training in this area as relevant and important to their work (9) and the scientific community shows an increase in published research on animal welfare over time (10–12). In addition, there is a growing public concern for animal welfare and an ethical requirement to comply with international standards and national regulations on zoo animal welfare (4, 13).

According to the Single Public Registry of Wildlife Operators (14), in Argentina there are 16 officially registered institutions that house wild fauna, with numerous populations of diverse native and exotic species, maintained under different conditions of animal welfare, and with dissimilar realities in terms of human and financial resources. In addition to the interest of researchers and zoo staff, the active demands of public opinion and animal rights NGOs have led to official interventions to initiate conversion processes in many zoos, with animal welfare as the main driver. It has also led to an update of national and territorial regulations, establishing animal welfare as a priority by applying the highest welfare standards for individuals, through adequate facilities and management modalities in zootechnical, ethological, sanitary, and genetic terms (15).

Ensuring animal welfare requires knowledge, experience, and institutional commitment, as well as the deployment of comprehensive and robust animal welfare assessment tools, which can be implemented at two levels: institutional (examining policies, resources, programs, and practices) or individual (providing an assessment of animals and their environments) (3, 16). As animal welfare is a multidimensional field of study (17–19), welfare assessment should consider multiple criteria (20–22), with a holistic evidence-based approach (3). Therefore, most welfare assessments strategically include animal-based measurements (ABM) that address aspects of the actual welfare state of the animals in terms of their behavior, mental state, health, and physical condition. They also incorporate resource-based (RBM) and management-based measurements (MBM) that can be correlated to ABM and used to identify risks for animal welfare and causes of poor welfare, so as to implement improvement strategies (23).

The approach in the construction of protocols to assess animal welfare, their methods, and the way in which they should be evaluated or validated depend on the goals, which need to be clearly defined before starting the development process. Botreau et al. (20) identify three main models for assessing animal welfare according to the intended goals: descriptive, normative, and prescriptive. The descriptive model is used to depict a pre-existing situation that is stable and independent of any observation, thus providing the ability to characterize and compare observed situations. The normative model explains how things should be or how people should act, and aims to provide evaluation procedures to verify the appropriateness of collected information in relation to predefined rules. Finally, the prescriptive approach does not assume any pre-existing situation to be described; it aims to collect and organize relevant information to facilitate the formulation of recommendations to achieve a goal.

Currently, there are different tools to assess animal welfare in zoos. Those that can be applied to multiple species usually consist of extensive checklists with questions aimed at revealing what the conditions of the physical and social environment are like and provide insight into the welfare of an individual animal [e.g., (3, 16, 24)]. Some of them also consider and integrate life stages, in relation to species and individual differences [e.g., (25)]. Although these protocols can be useful to easily improve animal welfare monitoring, they have some limitations, especially regarding the lack of guidance in the selection and interpretation of indicators, and thus, a non-tested reliability on applicants' criteria. Validated protocols also exist (21, 26–30), but they have been developed specifically for very few of the enormous variety of wild animal species that could require assessment (24).

This study aimed to develop, test in the field, and describe an animal welfare assessment protocol for wild animals under human care, which can be applied on a daily basis, noninvasively, and at a low cost, under the aforementioned prescriptive model. That is, first the current welfare status of the animal is assessed to understand the starting point and then its evolution is monitored by collecting information that allows the development of tailor-made recommendations and rapid decision making. Hence, it was intended that the protocol would be able to provide two types of assessment: comprehensive (whether initial diagnosis or in the face of important events, such as changes in the environment, group structure, and/or management) and regular (frequent monitoring to detect early deviations). Simultaneously, it aimed to obtain an intermediate solution between protocols that are easy to apply yet rely entirely on the judgment of the assessors, and validated but species-specific protocols that are useful only for assessing the species for which it was developed.

## Materials and methods

### Site

The protocol was tested at an Argentinean zoo, member of Asociación Latinoamericana de Parques Zoológicos y Acuarios (ALPZA) and World Association of Zoos and Aquariums (WAZA), which was in the process of transformation and restructuring. The protocol was applied and tested between October and December 2017.

### Elaboration of the protocol

The protocol was given the name Ackonc-AWA, which combines the purpose of conducting animal welfare assessments (AWA) with the role of the individuals involved in the observation and data collection process (hereafter, sentinels), given that the phonetics of the name reflects the native Andean word “ackoncahua” which is translated as “sentinel”.

The conceptual animal welfare framework adopted to create Ackonc-AWA protocol was the Five Domains Model (31), with a joint approach between the behavioral domain and the mental domain. Based on a literature review through research databases (PubMed and Google Scholar), with date restriction from January 2008 to July 2017, in English and Spanish, a selection of scientifically supported indicators previously used in welfare assessment protocols applied to farm, laboratory and zoo animals was obtained. Some of these indicators and their references were modified to adapt them to the characteristics of the zoo, to the variety and characteristics of species to be evaluated, and taking into account previous experiences of the researchers on animal welfare assessment in zoos. Interviews and meetings with personnel from different areas of the zoo were conducted. During the interviews, questions related to animal welfare were asked (e.g., When you observe the animals under your care, what do you look at? How do you notice if there is any discomfort, pain or something wrong with them?). Their responses were taken into account when selecting, eliminating, or adapting certain indicators in the protocol.

The principle of feasibility was taken into account for the selection of the welfare indicators (32, 33). The researchers also considered the need for the institution's own staff to be able to collect the data easily, subject to adequate training and performance evaluation. Thus, all measurements involving physical invasion or restraint of the animals, and indicators that require further laboratory analysis (e.g., metabolic profiling), were excluded. For this test, all the animal welfare assessments were performed hands-off, by remote observations at a distance.

In addition, two meetings were held with eleven representatives of different areas of the zoo (Veterinary, Nutrition, Biology, Behavior, Animal Care, Animal Welfare Management and Planning) to submit their input to a multi-disciplinary discussion in a focus group in order to select agreed upon items for assessment, as a way to provide content validity (13).

Once the first selection of the indicators to be assessed had been made, two types of forms were developed: an initial form and a follow-up form. The initial form consisted on 45 indicators (23 ABM, 19 RBM, and three MBM) (Table 1), which was meant to be carried out the first time an animal is assessed, and then on a semi-annual basis, or in the face of important changes in the environment, group structure and/or management of the animal under study. At this first step, the RBM and MBM were exhaustively considered together with ABM, to detect risk factors of poor welfare, even before the occurrence of identifiable manifestations by means of ABM. The follow-up form consisted exclusively of ABM (23 indicators) to facilitate the data collection process and reduce the time required to carry out the observations, and was intended to be applied daily or weekly. The frequency of use of the follow-up form can be adjusted according to need. As a starting point, the researchers suggest a weekly application. However, a higher frequency (i.e., daily) could be used for continuous monitoring of a newly moved animal or changes in group composition, management or enclosure characteristics to detect early alterations in ABMs that reflect a deterioration in welfare.

**Table 1:** List of indicators selected to test on-field reliability and feasibility, and sentinels assigned according to their availability, area of daily performance and experience.

	**Sentinels assigned to the on-field feasibility and reliability test of each indicator**						
	**External**	**Zoo staff (departments)**					
	**Researchers**	**V**	**N**	**Bi**	**Be**	**AC**	**AWMP**
**Nutrition domain**							
Body condition score (ABM)	Yes	Yes	Yes	No	No	Yes	Yes
Food intake (ABM)	Yes	Yes	Yes	No	No	Yes	Yes
Food availability (RBM)	Yes	No	Yes	Yes	No	Yes	Yes
Nutritional quality and safety of food (RBM)	Yes	No	Yes	No	No	No	Yes
Macroscopic condition of food (RBM)	Yes	No	Yes	No	No	No	Yes
Food presentation (RBM)	Yes	No	Yes	No	Yes	Yes	Yes
Water intake (ABM)	Yes	No	Yes	No	No	Yes	Yes
Availability of water (RBM)	Yes	No	Yes	Yes	No	Yes	Yes
Macroscopic quality of water (RBM)	Yes	No	Yes	No	No	Yes	Yes
Presentation of water (RBM)	Yes	No	Yes	Yes	Yes	Yes	Yes
**Environment domain**							
Substrate (RBM)	Yes	No	No	Yes	Yes	Yes	Yes
Temperature/humidity/ventilation (RBM)	Yes	Yes	No	No	No	Yes	Yes
Lighting (RBM)	Yes	Yes	No	Yes	No	Yes	Yes
Enclosure maintenance (RBM)	Yes	No	No	No	No	Yes	Yes
Enclosure hygiene (RBM)	Yes	No	No	No	No	Yes	Yes
Enclosure dimensions (RBM)	Yes	No	No	Yes	Yes	Yes	Yes
Environmental complexity (RBM)	Yes	No	No	Yes	Yes	Yes	Yes
Surrounding enclosures (RBM)	Yes	No	No	No	Yes	Yes	Yes
Shelter availability (RBM)	Yes	No	No	No	Yes	Yes	Yes
Public (RBM)	Yes	No	No	No	Yes	Yes	Yes
Group composition (RBM)	Yes	No	No	Yes	No	No	Yes
Environmental choice and control opportunities (RBM)	Yes	No	No	No	No	Yes	Yes
Management choice and control opportunities (MBM)	Yes	No	No	No	Yes	Yes	Yes
Environmental enrichment (MBM)	Yes	No	No	No	Yes	Yes	Yes
Training procedures (MBM)	Yes	No	No	No	Yes	Yes	Yes
**Health domain**							
Defecation behavior (ABM)	Yes	Yes	No	No	No	Yes	Yes
Stool score (ABM)	Yes	Yes	No	No	No	Yes	Yes
Micturition behavior (ABM)	Yes	Yes	No	No	No	Yes	Yes
Urine appearence (ABM)	Yes	Yes	No	No	No	Yes	Yes
Coat/feathers/tegument (ABM)	Yes	Yes	No	No	No	Yes	Yes
Lesions/injuries (ABM)	Yes	Yes	No	No	No	Yes	Yes
Hooves/claws/teeth (ABM)	Yes	Yes	No	No	No	Yes	Yes
Locomotion (ABM)	Yes	Yes	No	No	No	Yes	Yes
Sleep/wakefulness (ABM)	Yes	Yes	No	Yes	No	Yes	Yes
Signs of illness (ABM)	Yes	Yes	No	No	No	Yes	Yes
**Behavior and affective states domain**						
Reaction to strangers (ABM)	Yes	No	No	No	Yes	Yes	Yes
Interaction with zookeepers (ABM)	Yes	No	No	No	Yes	Yes	Yes
Exploration (ABM)	Yes	No	No	No	Yes	Yes	Yes
Social, affiliative and maternal-filial behavior (ABM)	Yes	No	No	No	Yes	Yes	Yes
Reproductive behavior (ABM)	Yes	No	No	Yes	Yes	Yes	Yes
Agonistic behavior (ABM)	Yes	No	No	No	Yes	Yes	Yes
Use of environmental enrichment (ABM)	Yes	No	No	No	Yes	Yes	Yes
Stereotypic behavior (ABM)	Yes	No	No	No	Yes	Yes	Yes
Behavioral diversity (ABM)	Yes	No	No	Yes	Yes	Yes	Yes
Space use (ABM)	Yes	No	No	No	Yes	Yes	Yes

V, Veterinary; N, Nutrition; Bi, Biology; Be, Behavior; AC, Animal Care; AWMP, Animal Welfare Management and Planning; ABM, Animal-based measurement; RBM, Resources-based measurement; MBM, Management-based measurements.

Additionally, a user's manual was written with instructions on the method used to assess and score each indicator. Both the indicators selected and the instructions for their assessment were the same for the different species and individuals included in the pilot test, although changes and clarifications were made in the user's manual to adapt them to the differential characteristics of each taxon.

Before beginning the assessment, sentinels had to be familiar with the following information about the species to be assessed: biological and behavioral features (including specie's ethogram); housing and handling requirements recommended by international associations; nutritional information (diet received by the animal or group being evaluated) and both routine and scheduled activities (e.g., feeding time, enclosure cleaning, training sessions, environmental enrichment, animal rotation and other interfering activities planned for the day of the assessment, such as capture for veterinary examination or transfer to another enclosure). Likewise, sentinels should have a layout/map and information about the location and dimensions of the enclosure.

Every effort should be made to minimize the impact of the presence of the sentinels on the behavior of the animal under study. Sentinels should remain out of sight and avoid any kind of interaction with the observed animal during the data collection to minimize the impact of his or her presence on the behavior of the animal under study (e.g., choosing an observation point to allow the sentinel to be as hidden as possible or remaining as long as necessary without interacting with the animal until it withdrew its attention from the sentinel's presence).

Indicators were rated on a 3-point scale (A—normal/no observable welfare risk; B—mild deviation/welfare risk; C—severe deviation/welfare risk). For indicators that could be rated in several contexts (e.g., animals that have access to different enclosures at different times), rating was made according to the context that represented a higher level of animal welfare compromise. When any indicator was rated “B” or “C”, the sentinel provided additional information about this on the “Notes” column.

### On-field feasibility test

#### Animals

The selection of the species and individuals on which the protocol was tested was based on the following inclusion criteria: 1—prospective permanence of the animals in the zoo: longer than 2 years; 2—easy identification: phenotypic characteristics or features that made it possible to individualize the animals housed in groups; and 3—include species from different taxonomic categories to test the ability of Ackonc-AWA protocol to be applied for different taxa. As a result, 14 individuals (ten mammals, two birds, and two reptiles) from different orders and families were selected (Table 2). Ackonc-AWA was also tested on one group of 12 capybara (*Hydrochoerus hydrochaeris*), to explore the potential usefulness of the protocol for group assessment, with proper modifications or adaptations.

**Table 2:** Information about the animals on which the Ackonc-AWA protocol was tested.

	**Family**	**Species**	**Gender**	**Age (Years)**	**Level of assessment**
**Order mammals**					
Primates	Hominidae	*Pan Troglodytes*	Male	11	Individual
		*Pongo* spp.	Female	31	
Carnivora	Canidae	*Chrysocyon brachyurus*	Female	16	
	Felidae	*Panthera tigris tigris*	Male	11	
	Otariidae	*Otaria flavescens*	Female	10	
Pilosa	Myrmecophagidae	*Myrmecophaga tridactyla*	Female	12	
Proboscidea	Elephantidae	*Elephas maximus*	Female	50	
Perissodactyla	Tapiridae	*Tapirus terrestris*	Male	10	
Artiodactyla	Camelidae	*Vicugna vicugna*	Male	13	
Rodentia	Caviidae	*Hydrochoerus hydrochaeris*	6 females 5 males 1 Unknown	11 adults 1 young	Individual and Group
**Order birds**					
Cathartiformes	Cathartidae	*Vultur gryphus*	Male	10	Individual
Psittaciformes	Psittacidae	*Anodorhynchus hyacinthinus*	Female	27	
**Order reptiles**					
Testudines	Chelidae	*Acanthochelys spixii*	Female	4	Individual
Squamata	Teiidae	*Salvator rufescens*	Male	8	

#### Sentinels

Sentinels assigned to observe, record and score the indicators on site were selected from the different areas involved in animal management and care, based on interviews, in search of those who met a combination of experience, training, predisposition and observation skills. Their election was also agreed with representatives of the institution in order to avoid hindering or disrupting daily activities. Hence, the team of sentinels consisted of a group of three external veterinarians experienced in animal welfare assessments (the first three authors of this work, hereafter the researchers) and a group of nine zoo staff members with no prior experience in animal welfare assessments, belonging to different departments [one from Veterinary, one from Nutrition, one from Biology, two from Behavior, two from Animal Care (zookeepers) and two from Animal Welfare Management and Planning (AWMP)].

All inexperienced sentinels received a 4 h theoretical and practical training on animal welfare assessment in general and on the use of the protocol in particular, designed and delivered by the researchers. A virtual library was also created with ethograms and information on each of the 14 species' nutritional, physiological, environmental and behavioral needs, selected by the researchers from books, husbandry manuals and peer-reviewed scientific publications. All sentinels were given access to this virtual library and were instructed to read the documents selected for the corresponding species before beginning the on-field feasibility test.

The researchers and the zoo staff from the AWMP Department were exclusively dedicated to this task, so they evaluated the entire protocol (all indicators). On the other hand, the rest of the sentinels were assigned a different number of indicators to score, since they had different availability to collaborate with this research (Table 1). For the latter group, indicators would be scored during the zoo routine schedule and with minimum interference to the daily management and procedures. Likewise, the assignment of the indicators to be rated was made considering their area of daily performance and previous experience. For instance, health-related indicators were assigned to the zoo veterinarian, and nutrition-related indicators to the nutrition expert.

#### Test-retest reliability

Three sentinels were assigned the assessment of the same animal at two different time points. Test-retest agreement rate was corrected for chance by kappa statistics (34). Inter-observer reliability could not be assessed due to the limited availability of zoo staff involved in this pilot test. The statistical processing of the data was carried out using the software Infostat^®^ (35) and VassarStats: Website for Statistical Computation (36).

#### Feasibility

##### Completeness of the forms

For the animal welfare assessment to be comprehensive, all indicators in Ackonc-AWA must be completed, except for those that do not apply to a given species, due to its particular nature (e.g., water consumption in underwater species) or under specific situations (courtship behavior outside the reproductive season). In such cases, sentinels were instructed to use the legend “does not apply” to differentiate them from those that could be left blank due to other reasons (e.g., lack of time, impossibility of taking the measurement, not provided access/information).

The average completeness of the forms was determined by averaging the degree of completeness achieved by all sentinels for all species. In addition, a ranking of the indicators most often left blank was made by counting the number of times that each indicator was not evaluated when it should have, in relation to the total of forms (both initial and follow-up) across species and sentinel groups.

##### Degree of difficulty represented by the observation and recording process

Ackonc-AWA protocol was designed so that the observations and completion of the forms can be done by the zookeepers, combining this activity with their other responsibilities. Therefore, it was important to determine the degree of difficulty perceived by the staff in applying the chosen indicators. For this purpose, after completing the Ackonc-AWA forms, each sentinel was asked to assign a degree of difficulty to fill out each form between 1 and 10, with 1 being the minimum and 10 the maximum. At the bottom of each form, the sentinels had to specify which indicator was found as the most difficult to evaluate. With these responses, the indicators were rated for their level of difficulty, from the most often reported to the least often reported. The results were analyzed by averaging the degree of difficulty assigned for all species and sentinels, differentiating between initial and follow-up assessment forms.

##### Time required to complete the forms

It was intended that the Ackonc-AWA protocol require <2 h per individual or group for data collection since long application protocols have more difficulties to be used regularly in zoological institutions, especially in those lacking resources or exclusive personnel for this purpose, a very frequent situation in Latin America. The average time (in minutes) required to complete the two welfare assessment protocol forms was recorded for all species and sentinels, differentiating between initial and follow-up assessment forms.

Ethical review of the project was requested to the Institutional Committee for the Care and Use of Laboratory Animals (CICUAL) of the Faculty of Veterinary Sciences of the University of Buenos Aires, and a review exemption was granted given the observational nature of the project. The study focused on the non-invasive/intrusive assessment of animal welfare, so no interventions of any kind were carried out on the animals. There were no potential adverse effects, nor foreseeable risks or hazards associated with this project, with regards to animal, plant and/or human wellbeing. The participation of zoo staff in this study was completely voluntary and under written informed consent. The survey responses were strictly confidential and data from this research was reported only in the aggregate. The information was coded and remains confidential.

## Results

### On-field feasibility test

#### Test-retest reliability

The mean intra-observer proportion of agreement was 0.8652 among the sentinels and 0.8678 among indicators (ABM, RBM, and MBM). The mean observed Kappa was 0.7763 among the sentinels and 0.7681 among indicators, which on the Landis and Koch (34) scale is substantial agreement (Table 3). Although Cohen's test ruled out a random component, more trials are needed to increase the statistical power of the test.

**Table 3:** Test-retest reliability calculated for the on-field test of Ackonc-AWA protocol.

	**Cohen's kappa**	**SE**	**CI95%**	**Proportion of agreement**	***n***
**Sentinels**					
1	0.7391	0.0798	(0.5827; 0.8955)	0.8421	57
2	0.7863	0.0821	(0.6255; 0.9471)	0.8846	52
3	0.7997	0.0623	(0.6777; 0.9217)	0.8696	69
Overall pondered	0.7763			0.8652	
**Indicators**					
ABM	0.7574	0.0759	(0.6086; 0.9062)	0.8816	76
MBM	0.6774	0.2040	(0.2775; 1)	0.8000	10
RBM	0.7877	0.057	(0.6761; 0.8993)	0.8636	88
Overall pondered	0.7681			0.8678	

#### Feasibility

##### Completeness of the forms

The average completeness for the initial form was 86.21% whereas for the follow-up form it was 79.07%. The top ten indicators most often left blank were part of both assessment forms and were therefore analyzed together. No indicators were left blank over 50% of the times. Only Water intake was left blank over 40% of the times (56 times; 42.10%). Three indicators were left blank between 40 and 30% of times: Micturition behavior (44 times; 33.08%), Defecation behavior (42 times; 31.58%), and Use of environmental enrichment (42 times; 31.58%); two indicators were left blank between 30 and 20% of times: Social behavior (37 times; 27.82%) and Reproductive behavior (30 times; 22.56%); and four indicators were left blank between 20 and 10% of times: Hooves/claws/teeth (24 times; 18.04%), Agonistic behavior (20 times; 15.04%), Food intake (17 times; 12.78%) and Behavioral diversity (16 times; 12.03%). The rest of the indicators included in the protocol were left blank <10% of the time.

##### Degree of difficulty represented by the observation and recording process

The mean reported difficulty across species and sentinels was 4.79 +/– 1.13 for the initial form and 5.20 +/– 1.51 for the follow-up form. Analysis of sentinel responses showed that the indicator most frequently reported as difficult to assess was Behavioral diversity (54 times; 40.60%) followed by Defecation behavior (31 times; 23.31%), Micturition behavior (29 times; 21.80%), Hooves/claws/teeth (13 times; 9.77%), Water intake (12 times; 9.02%) and Food intake (11 times; 8.27%).

##### Time required to complete the forms

The average time across species and sentinels required to complete the initial form was 51.32 min. +/– 29.36 min. Completion of the follow-up form took an average of 58.32 min +/– 23.11 min. Table 4 shows the amount of time (in minutes) required to complete initial and follow-up welfare assessment forms of Ackonc-AWA protocol for each of the species included in the study. Activity budget sheets were later added to the protocol (see below).

**Table 4:** Time in minutes (mean +/– SD) required to complete initial and follow-up welfare assessment forms of Ackonc-AWA protocol.

**Species**	**Initial form**		**Follow-up form**	
	**Mean**	**SD**	**Mean**	**SD**
*Pan Troglodytes*	56.4286	28.9704	86.2500	22.5000
*Pongo* spp.	46.0000	29.6648	40.0000	0.0000
*Chrysocyon brachyurus*	47.8571	35.1019	31.2500	6.2915
*Panthera tigris tigris*	60.0000	46.9042	40.0000	28.2843
*Otaria flavescens*	66.6667	37.7712	37.5000	9.5743
*Myrmecophaga tridactyla*	29.0000	20.7364	67.5000	74.2462
*Elephas maximus*	46.0000	25.8360	75.0000	32.7872
*Tapirus terrestris*	55.0000	27.3861	87.5000	12.5831
*Vicugna vicugna*	30.0000	7.0711	50.0000	29.4392
*Hydrochoerus hydrochaeris*	63.2500	21.1213	58.2222	15.8096
*Vultur gryphus*	90.0000	42.4264	63.7500	25.6174
*Anodorhynchus hyacinthinus*	36.6667	23.5938	61.7500	13.3760
*Acanthochelys spixii*	41.6000	45.8290	43.7500	9.4648
*Salvator rufescens*	50.0000	18.7083	74.0000	43.6119
Total	51.3192	29.3658	58.3194	23.1132

### Structure of Ackonc-AWA and application guidelines/criteria

In the face of on-field feasibility results, some changes were implemented for the assessment of the indicator “Behavioral diversity” within the Behavioral and mental domain by the introduction of activity budget sheets of 20 min each, on three (or four, when possible) different time slots (see Supplementary Table 1). After sentinels complete the activity budgets sheets, a trained analyst (external or personnel of the institution) should evaluate the data and assign the appropriate score (A, B or C) for the indicators “Behavioral diversity” and “Space use”.

No changes were made for the indicators included in the Nutritional, Environmental and Health domains. As a result, a total of 45 indicators (23 ABM, 19 RBM, and three MBM) were selected to integrate Ackonc-AWA, covering the five animal welfare domains.

#### Nutritional domain

Three ABM (Body condition score, Food intake, and Water intake) and seven RBM (Food availability, Nutritional quality and food safety, Macroscopic condition of food, Food presentation, Availability of water, Macroscopic quality of water, and Presentation of water) were selected to assess the nutritional domain. Table 5 summarizes the methods, references, and scoring system required for this purpose.

**Table 5:** Summary of the most relevant information that is provided in the user's manual for assessing nutritional domain.

**Indicator**	**Method**	**Reference**	**Scoring**
Body condition score (ABM)	It shall be assessed visually. Only when there is no risk for humans or animal's welfare, it may also be assessed by palpation. Use a standardized 5-point scale scientifically validated for the species under study.	Does the animal have a body condition appropriate to their species, age, sex and physiological state?	A: 3, B: 2 o 4, C: 1 o 5. In case B or C, clarify in “observations” to which BCS it corresponds.
Food intake (ABM)	Observe the eating behavior and the daily amount of food consumed.	Is the feed intake adequate for the animal according to their age, sex, physiological state and health condition?	A: normal appetite. B: hyporexia, pica, trichophagia, coprophagia (In some species coprophagia is not pathological). C: anorexia, polyphagia or any type of disturbance that is not allowing adequate food intake (even if appetite is not affected).
Food availability (RBM)	Observe the time at which food is offered in the indoor and outdoor enclosures (features to consider: number, competition for access, location and height, cleanliness and maintenance condition of feeders or feeding zone).	Is the food available and sufficient considering age, sex, physiological state and health condition of the animal?	A: all the features are adequate. B: only one of the features is not adequate, but it does not prevent access to the food. C: the food is not accessable and/or two or more features are not adequate.
Nutritional quality and safety of food (RBM)	Request information from the nutrition department. If possible, send food samples for analysis. Relevant literature should be used to obtain information on the reference values and analyses required for the species under study.	Is the diet adequate in nutrients (according to the species, age, physiological and health status) and are the ingredients safe and secure (free of contaminants and toxins, cold chain mantained)?	A: the diet is adequate, safe and secure. C: either nutrient profile or food safety criteria is not adequate.
Macroscopic condition of food (RBM)	Observe the food offered to the animal (alterations to consider: bruises, insects, mold, rotting, fruit ripening, fecal matter mixed with the food).	Is the food offered to the animal in good condition?	A: no alterations are observed. B: only one food or portion have only one of the mentioned alterations. C: one or more foods or portions have two or more of the mentioned alterations.
Food presentation (RBM)	Observe and compare the way in which the food is presented in the zoo with how it is found in the evolutionary environments of the species (features to consider: frequency, portion size, timing, texture, consistency, temperature and location).	Does the presentation of the food respect the way the species feeds in the wild?	A: all features to be considered are adequate. B: only one of the features is not adequate, but it does not impede the ingestion of food. C: two or more features are inadequate
Water intake (ABM)	Observe the drinking behavior and the daily amount of water consumed.	Does water consumption match the animal's requirements?	A: normal intake. B: slight increase or decrease in water intake unrelated to weather conditions. C: significant increase or decrease in water intake unrelated to weather conditions and/or difficulty in swallowing or ingesting water.
Availability of water (RBM)	Observe the water troughs and other water sources in the indoor and outdoor enclosures (features to consider: number, competition for access, location and height, cleanliness and maintenance).	Is the animal provided with sufficient and accessible water at all times?	A: all features to be considered are respected. B: only one of the features to be considered is not respected, but it does not prevent access to water. C: water is not accessible and/or two or more features to be considered are not respected.
Macroscopic quality of water (RBM)	Observe the water offered to the animal (features to consider: color, odor, presence of food debris and other visible particles, greenery)	Is the water offered to the animal in good condition?	A: all features to be considered are adequate. B: only one of the features to be considered is not adequate, but it does not prevent the ingestion of water. C: two or more features to be considered are not adequate.
Presentation of water (RBM)	Observe and compare the way in which water is presented in the zoo with how it is found in the evolutionary environments of the species and their drinking behavior.	Does the presentation of water respect the way it is found in the wild and accordingly with the species drinking behavior?	**A:** the presentation of water respects the way the species drinks in the wild. **B:** the presentation of water partially respects the way the species drinks in the wild (if the species has more than one way of drinking water, its presentation does not allow to express at least one of them) **C:** the presentation of water does not respect the way the species drinks in the wild.

ABM, Animal-based measurement; RBM, Resources-based measurement.

#### Environmental domain

Twelve RBM (Substrate, Temperature/humidity/ventilation, Lighting, Enclosure maintenance, Enclosure hygiene, Enclosure dimensions, Environmental complexity, Surrounding enclosures, Shelter availability, Public, Group composition, Environmental choice, and Control opportunities) and the three MBM (Management choice and control opportunities, Environmental enrichment, and Training procedures) were adopted. Table 6 summarizes the most relevant information provided in the user's manual for assessing environmental domain. To this end, sentinels had to be able to access and consider all areas destined to the animal (e.g., exhibitors, sleeping quarters, pens, handling areas, etc) to rate each indicator according to the sector(s) that imply a greater compromise to the welfare of the animal (or group).

**Table 6:** Summary of the information provided in the user's manual for assessing environmental domain.

**Indicator**	**Method**	**Reference**	**Scoring**
Substrate (RBM)	Observe the substrate of the enclosure and compare it with the typical natural environment of the species (features to consider: level of compaction, texture, hardness and temperature of the material, undulations and unevenness). If available, check the reference substrate requirements for the species in the husbandry manual.	Is the substrate suitable for the animal to rest comfortably and exhibit species-specific behaviors?	A: the substrate is suitable for the animal to rest comfortably and deploy species-specific behaviors. B: the substrate is inappropriate for the animal to rest comfortably or may prevent the manifestation of any species-specific behaviors. C: the substrate is inappropriate for the animal to rest comfortably and/or could prevent the manifestation of several species-specific behaviors.
Temperature/humidity/ ventilation (RBM)	Observe the conditions offered in the enclosure and compare them with the climatic characteristics of the ancestral environment of the species (features to consider: sources of heat or cold, shade and sun, and bathing facilities (e.g., water, mud or other). If the enclosure has a controlled system for temperature, humidity and ventilation, or if you have a device to measure these parameters, check and record the values. If available, check the reference temperature, humidity and ventilation requirements for the species in the husbandry manual.	Are the enclosure conditions adequate to allow the animal to maintain thermal comfort?	A: the enclosure presents conditions that allow maintaining an adequate thermal comfort in all its aspects. B: one of the aspects of the enclosure is deficient to maintain adequate thermal comfort without threatening the life of the animal. C: two or more of the aspects of the enclosure are deficient to maintain adequate thermal comfort, or only one aspect is deficient in a way that puts the animal's life at risk.
Lighting (RBM)	Observe the lighting of the enclosure and compare it with the typical natural environment of the species. If available, check the reference lighting requirements for the species in the husbandry manual.	Does the lighting in the enclosure respect the circadian cycle, the number of hours of light/darkness characteristic of the natural environment of the species and does it not affect or hinder vision or generate somatic disorders? Is the amount of sunlight entering the enclosure adequate according to the characteristics of the natural environment of the species?	A: natural and artificial lighting is suitable for the species. B: one of the components to be considered is not appropriate, without putting the animal's life at risk. C: two or more of the components to be considered are deficient, or only one is deficient but puts the animal's life at risk.
Enclosure maintenance (RBM)	Observe the maintenance conditions of the enclosure (features to consider: defects in the structure of the enclosure that may cause damage to the animals, poisonous plants within reach, exposure to electrical appliances or poorly protected electrical outlets, vegetation that could fall and cause damage, entry of disease-carrying animals or pests such as rodents).	Does the condition in which the enclosure is maintained pose no risk to the health and welfare of the animal or third parties?	A: the enclosure is in good maintenance conditions. B: there are some defects in the maintenance of the enclosure, which do not directly endanger the lives of animals or people. C: there are many defects in the maintenance of the enclosure and/or the defective feature(s) put the life of animals or people at direct risk.
Enclosure hygiene (RBM)	Observe the hygiene of the enclosure (features to consider: spoiled food, stagnant water, accumulation of feces and urine and dead animals). Consider that excess hygiene can also be detrimental (inadequate for the species or in higher concentration than recommended or with higher frequency than recommended). If available, check the recommended chemical types, concentration and frequency for the species (husbandry manual).	Is the enclosure maintained in adequate hygienic conditions? Are the chemicals used adequate in type and concentration? Is the frequency of cleaning adequate?	A: The enclosure is in good hygienic conditions and the cleaning routine is adequate for the species. B: there are some defects in the hygiene of the enclosure, which do not put the health of animals or people at direct risk. C: there are many defects in the hygiene of the enclosure and/or the defective feature(s) puts the health of animals or people at direct risk.
Enclosure dimensions (RBM)	Request the enclosure outline and verify that the declared dimensions match the actual dimensions. Take the necessary measurements and record the dimensions of the enclosure. When answering the reference question consider that the animal should be able to express the full repertoire of locomotor movements of their species, including running, climbing, flying or swimming at speed. If more than one individual is housed in the same enclosure, consider the number of animals per surface area. If available, check the reference requirements for the species (husbandry manual).	Do the dimensions of the enclosure allow the animal to move freely? Do they comply with the minimum space requirements stated in the husbandry manuals per individual?	A: the dimensions comply with existing recommendations and are adequate for the animal to move freely and express the full locomotor repertoire of its species. B: the dimensions allow the animal to move freely but hinder the expression of the full locomotor repertoire of its species and are below those recommended. C: dimensions do not allow the animal to move freely and/or impede the expression of the full locomotor repertoire of its species and are below those recommended.
Environmental complexity (RBM)	Observe the disposition of different areas and elements within the enclosure. Consider feeding and elimination zones, characteristics of the environment, land/water/air space ratio, implements for the vertical use of space. For an accurate evaluation of welfare it is essential to distinguish it from environmental enrichment.	Does the design of the enclosure allow for species-specific behaviors as well as differential use of each part of the space?	A: the design of the enclosure allows for differential use of each part of the space as well as the occurrence of all species-specific behaviors. B: the design of the enclosure allows differential use of each part of the space as well as the occurrence of most species-specific behaviors. C: the enclosure design does not allow differential use of each part of the space and/or prevents the occurrence of several of the species-specific behaviors.
Surrounding enclosures (RBM)	Observe the surrounding enclosures (features to consider: presence of visual barriers, pray, predators or competitors housed in adjacent enclosures and distance between enclosures).	Does the housing layout and design minimize stressful situations with animals in adjacent enclosures or loose animals?	A: the layout and design of the housing are adequate to minimize stressful situations with animals in adjacent enclosures or loose animals. B: only one of the features to be considered is deficient. C: two or more of the features to be considered are deficient.
Shelter availability (RBM)	Observe the existence, availability and adequacy of shelters for various weather conditions.	Do the animals have shelters to protect them from adverse weather conditions?	A: shelters provide full protection from inclement weather. B: shelters provide partial protection from inclement weather. C: shelters do not provide protection from inclement weather or there is no shelter or repair.
Public (RBM)	Observe the possibility of hiding from the public (features to consider: visual barriers, impediments for direct contact; first, second and third level barriers; free access to confinement areas).	Does the housing layout and design minimize stressful situations for the animal generated by the public?	A: the layout and design of the housing are adequate to minimize stressful situations with humans. B: only one of the features to be considered is deficient. C: two or more of the features to be considered are deficient.
Group composition (RBM)	Observe group composition, ALWAYS record in “observations”: number of adults (clarifying the sex of each one), juveniles (sex) and young, number of species and individuals in the same enclosure. If the enclosure is shared with another species, consider if this association is adequate for the species you are working with. (Features to consider:gregarious/solitary, number of individuals, proportion of males/females and offspring)	Is the group composition representative of the species?	A: the composition of the group is representative of the species in all features. B: the gregarious/solitary condition of the species is respected but one or more of the other features to be considered is deficient. C: the gregarious/solitary condition of the species is not respected and/or two or more of the other features to be considered are deficient.
Environmental choice and control opportunities (RBM)	Examine the enclosure and assess whether it offers the animals opportunities for control and choice. Consider: opportunities for choice of display or concealment, shade or sun, heat or cold, companionship or solitude, need to alternate exit to the main exhibit, access to the main exhibit during peak periods of the day—species with nocturnal or crepuscular habits, isolation from stressors derived from cleaning, maintenance and repair maneuvers.	Does the enclosure design allow the animal to choose where to be or what to do 24 h a day?	A: the design of the enclosure allows the animal to choose where to be or what to do, in all its aspects, during 24 h of the day. B: the enclosure design allows the animal to choose where to be or what to do, in various aspects, during at least the most active period of the day for the species. C: the enclosure design allows the animal to choose where to be or what to do in few or none of its aspects and/or opportunities for choice and control are present only during the period of the day of least activity for the species.
Management choice and control opportunities (MBM)	Interview staff and assess whether the management offers animals opportunities for control and choice. Consider all the aspects mentioned in “Environmental choice and control opportunities”	Does management allow the animal to choose where to be or what to do 24 h a day?	A: management allows the animal to choose where to be or what to do, in all its aspects, 24 h a day. B: management allows the animal to choose where to be or what to do, in several of its aspects, during at least the most active period of the day for the species. C: management allows the animal to choose where to be or what to do, in few or none of its aspects, and/or opportunities for choice and control are present only during the period of the day of least activity for the species.
Environmental enrichment (MBM)	Interview staff, check documentary records and verify the implementation of an appropriate and comprehensive environmental enrichment (EE) program. Consider anything that is not fixed or does not remain the same in the animal's environment, but can be placed and removed on a daily basis. For an accurate evaluation of welfare it is essential to distinguish it from environmental complexity.	Is there a formal, written EE program in place and implemented to promote species-specific behavioral opportunities and psychological well-being? Does it include nutritional, social, sensory, cognitive, and occupational environmental enrichment?	A: an EE plan/program is implemented according to a formal, written outline that promotes behavioral opportunities and psychological well-being and all steps are followed, including analysis of the animal's response to EE, as well as the various types of EE. B: an EE plan/schedule is implemented but no observation or analysis of the animal's response to EE is performed, or EEs does not go through an approval process from all areas (veterinary, biology, behavior, nutrition and keepers), or any of the types of EE mentioned in the question are not implemented. C: no EE is performed or it is only performed by the individual will of the keeper or volunteers, without an official plan by the institution.
Training procedures (MBM)	Interview staff, check documentary records and verify the implementation of a comprehensive and appropriate training plan.	Is there a formal, written animal training plan in place for the animal?	A: training for veterinary and handling maneuvers, cognitive enrichment, strengthening of the human-animal bond and animal exercise is carried out by duly trained personnel, using validated techniques, with a formal, written plan, and in the case of dangerous species, without direct contact between trainer and animal. B: only training for veterinary and handling maneuvers is carried out by duly trained personnel, using validated techniques, without direct contact between trainer and animal in the case of dangerous species, with a formal, written plan. C: training is not carried out and/or is carried out by inadequately trained personnel and/or by means of techniques with doubtful results and/or with direct contact between trainer and animal in the case of dangerous species and/or without a formal and written plan.

RBM, Resources-based measurement; MBM, Management-based measurements.

#### Health domain

Ten ABM (Defecation behavior, Stool score, Micturition behavior, Urine appearance, Coat/feathers/tegument, Lesions/injuries, Hooves/claws/teeth, Locomotion, Sleep/wakefulness, and Signs of illness) were selected to assess the health domain. Table 7 summarizes the most relevant information provided in the user's manual for assessing health domain.

**Table 7:** Summary of the information provided in the user's manual for assessing health domain.

**Indicator (ABM)**	**Method**	**Reference**	**Scoring**
Defecation behavior	If the animal is observed during defecation, check body posture, facial expressions and vocalizations.	Does the animal have difficulty or pain during defecation?	A: absence of difficulty or pain during defecation. B: slight difficulty or pain during defecation. C: difficulty or moderate to severe pain during defecation.
Stool score	Observe the characteristics of stool with the aid of the approved fecal condition scales for the species.	Is the stool adequate in terms of consistency, shape, color, frequency of excretion and macroscopic composition (blood, mucus, undigested food, foreign matter)?	A: normal stool, without alterations in any of the aspects to be considered. B: stool with some of the aspects to be considered slightly or incipiently altered. C: stool with some of the aspects to be considered severely altered.
Micturition behavior	If the animal is observed during urination, check body posture, facial expressions and vocalizations.	Does the animal have difficulty or pain to urinate?	A: absence of difficulty or pain on urination. B: slight difficulty or pain during urination. C: difficulty or pain moderate to severe pain during urination.
Urine appearance	Observe the characteristics of urine such as stream fluidity, urine color, frequency and quantity.	Are there any abnormalities in the urine?	A: normal urine, without alterations in any of the aspects to be considered. B: urine with some of the aspects to be considered slightly or incipiently altered. C: urine with two or more of the aspects to be consider altered in a severe way or for several days.
Coat/feathers/ tegument	Observe the characteristics of the skin and the phanerae (features to consider: quantity, brightness and integrity).	Is the plumage/fur/coat/ integument in good condition?	A: good condition of plumage/coat/integument. B: Slight alteration in the quantity or condition of the condition of the coat/plumage/tegument without alteration of its integrity. C: severe alteration in the quantity or condition of the coat/plumage/tegument.
Lesions/injuries	Note the presence of wounds (Pay attention to hair removal, abrasion, redness, swelling, bleeding, abscesses, bruises, presence of flies).	Does the animal appear free of lesions or wounds?	A: absence of lesions and wounds. B: shallow wounds or lesions, small in size and low in number, without infection, suppuration or flies, with mild and short-term effects on animal welfare. C: deep, medium or large wounds or lesions, several in number, with infection, suppuration or flies, with moderate to severe or long-term effects on animal welfare.
Hooves/claws/teeth	According to the species, observe the condition of hooves, claws and teeth as appropriate. Take advantage of situations where the animal is close enough to inspect them (e.g., in training sessions for clinical procedures, when performed).	Is the animal free of overgrowth or lesions on hooves, nails, claws, teeth?	A: hooves/claws/teeth are free of overgrowth and lesions. B: hooves/claws/teeth show mild to moderate overgrowth but are free of lesions. C: hooves/claws/teeth show severe overgrowth and/or lesions.
Locomotion	Observe how the animal moves around the enclosure (features to consider: lameness, reluctance to walk or jump, facial expressions of pain and/or vocalizations while moving)	Does the animal ambulate without difficulty?	A: the animal moves without difficulty or evidence of pain. B: the animal presents mild lameness (grade 1 or 2). C: the animal presents moderate to severe lameness (grade 3 or 4) and/or is reluctance to move and/or experiences evident pain when walking.
Sleep/wakefulness	Observe sleep and activity behaviors at different times of the day.	Does the animal show activity in accordance with the circadian rhythm of its species in nature?	A: the animal's activity is in accordance with the circadian rhythm of the free-living species. C: the animal does not present an activity in accordance with the circadian rhythm of the free-living species.
Signs of illness	Look for any signs of disease (pay attention to ears, mouth, muzzle, perineal region, respiration, general condition and other anatomical regions or body structures where signs of disease may be evident, depending on the species). Consider signs of disease as identified in the available literature for the species.	Does the animal appear healthy and free of visible signs of disease?	A: the animal appears clinically healthy. B: mild and/or recent symptoms of disease, with minimal effect on animal welfare and/or good prognosis. C: moderate or severe symptoms of disease, or mild but long-standing symptoms, with significant effects on animal welfare and/or unfavorable prognosis.

ABM, Animal-based measurement.

Given the multispecies purpose of the Ackonc-AWA protocol, it is important to note that for some species (e.g., reptiles, birds) it may be necessary to score the indicators “Defecation behavior” and “Stool score”, together with “Micturition behavior” and “Urine appearance” respectively, due to their physiologic and anatomic features.

#### Behavioral and mental domain

Ten ABM (Reaction to strangers, Interaction with zookeepers, Exploration, Social, affiliative and maternal-filial behavior, Reproductive behavior, Agonistic behavior, Use of environmental enrichment, Stereotypic behavior, Behavioral diversity, and Space use) were selected to assess the Behavioral and mental domains. Table 8 summarizes the most relevant information provided in the user's manual for assessing behavioral and mental domains, through ten ABM.

**Table 8:** Summary of the most relevant information that is provided in the user's manual for assessing behavior and mental domain indicators.

**Indicator (ABM)**	**Method**	**Reference**	**Scoring**
Reaction to strangers	It should be assessed at any time when the public or zoo staff are unfamiliar to the animal. Assess whether the presence of strangers modifies the occurrence or development of species-specific behaviors, or if signs of fear (e.g., hiding), agonism (e.g., stalking), or habituation (e.g., begging for food or actively seeking interaction) are observed.	Is the animal indifferent to the presence of the public, unfamiliar staff, or observers (if they are not people with whom it has daily contact)?	A: indifferent or positive. C: fear, hidding, aggressiveness, freezing.
Interaction with zookeepers	It should be evaluated any time the animal is in the presence of its keepers. Assess whether this presence modifies the occurrence or development of species-specific behaviors, or if signs of fear (e.g., hiding), agonism (e.g., stalking) or social behaviors (e.g., asking for petting or actively seeking interaction) are observed.	Does the animal have a positive relationship with their keepers?	A: alert, responds to call and commands. B: indifference. C: fear, agonistic behavior.
Exploration	Observe the animal's active exploration of its environment (consider that in addition to wandering, the individual listens, sniffs, licks, or manifests any other component of species-typical exploratory behavior).	Does the animal roam the enclosure and its sorroundings directing their senses to relevant stimuli?	A: exploration is observed. B: exploration is only observed in response to novel stimuli (e.g., environmental enrichment). C: no exploration is observed.
Social, affiliative and maternal-filial behavior	Observe affiliative bonds, such as nurturing and maternal-filial relationship, grooming sessions, or any other component of species-typical social behavior). If the animal is housed in solitary, observe if there are interactions with animals from adjacent enclosures.	Does the individual interact with others in a positive way?	A: positive interaction with other animals. B: indifference or isolation. C: aggressiveness, fear.
Reproductive behavior	Observe the occurrence of reproductive behavior according to the time of year (and species characteristics), proximity of individuals of the same species and different sex, presence of young, courtship behaviors (depending on species: sniffing, urination, marking spray, vocalizations, sensory orientation, etc.). Consider these factors in the different possible contexts (e.g., animals housed in the same enclosure, animals housed in adjacent enclosures with different possibilities of direct contact, and animals housed in nearby enclosures but without direct contact).	Does the animal deploy species-specific reproductive behavior?	A: appetitive and consummatory phases of reproductive behavior are observed in animals housed in the same enclosure during the breeding season. B: incomplete repertoire of reproductive behavior (e.g., substitution behaviors or blank firing) are observed in the breeding season. C: absence of reproductive behavior during the breeding season.
Agonistic behavior	Observe for agonistic interactions and weigh the results. If the animal is housed alone, observe for interactions with animals in adjacent enclosures. Specify in “observations” which individuals are involved and the observed behavior.	Do animals interact with others of the same or related species in a negative way?	A: no more than 3 agonistic interactions marked on the time budget sheets during the 60 min and NO obvious negative effects on animal welfare (e.g., moderate to severe injury or wounding). C: 4 or more agonistic interactions marked on the time budget sheets during the 60 min or < 4 WITH obvious negative effects on animal welfare.
Use of environmental enrichment	Evaluate the animal's response to environmental enrichment (EE) by direct evidence (DE) (visualization of the animal interacting with EE, observing it at the time it is offered) or by indirect evidence (IE) (visualization of the EE or its remains after the animal interacted—or not—with it). Clarify in “observations” which type of EE was observed during assessment.	Is there evidence of interaction with EE?	A: 5 (DE) or 3 (IE), B: 2, 3 or 4 (DE) or 2 (IE), C: 1 (DE or IE).
Stereotypic behavior	Observe for the presence of repetitive, unvarying behaviors with no obvious functional goals. In case B or C, describe in “observations” the behavior in question as detailed as possible.	Does the animal show any abnormal repetitive behavior?	A: the animal does not deploy repetitive behavior. B: repetitive behavior occurs but the pattern retains some variability (it does not always move the same body parts in the same way, it can do it with some variants) and low repeatability (no more than 5 repetitions in a row without stopping). C: the behavior has no variability (always moves the same body parts in the same way) or high repeatability (more than 5 repetitions in a row without stopping).
Behavioral diversity	Complete the “time and space budget sheets” provided by the analyst, in different time slots (morning, noon, afternoon and evening) as specified in the user's manual. Attention! The observer should not assign a score for this indicator. The analyst will be the one to assign the score in consideration of the richness of the behavior (number of behaviors) as well as the uniformity (frequency of each behavior) following the Activity budget method (37).	Does the animal perform species-specific behaviors at natural frequencies and appropriate diversity?	A: Time budget reflects 100% coverage of the functional categories, with no deviations in their proportion as expected for the species. B: the time budget reflects a coverage of between 70 and 100% of the functional categories, with slight deviations in their proportion according to what is expected for the species. C: Time budget reflects a coverage of < 70% of the functional categories, with marked deviations in their proportion according to what is expected for the species.
Space use	Complete the “time and space budget sheets” provided by the analyst, in different time slots (morning, noon, afternoon and evening) as specified in the user's manual. Attention! The observer should not assign a score for this indicator. The analyst will be the one to assign the score.	Does the animal make full use of the available space?	A: uses between 85 and 100% of the areas to which it has access. B: uses between 50 and 84% of the sectors of the enclosure to which it has access. C: uses between 0 and 49% of the sectors of the enclosure to which it has access.

ABM, Animal-based measurement.

## Discussion

This study introduced an innovative multi-species animal welfare assessment protocol for wild animals under human care, intended to overcome the use of generic welfare checklists and offer an alternative to challenging and time consuming species-specific tools (24). Ackonc-AWA protocol has several features in common with those of Kagan et al. (16), Brando and Buchanan-Smith [264], Sherwen et al. (3), and Ward et al. (24). They all cover the five domains of animal welfare (31), through indicators that provide information on physical, environmental, behavioral, and social state, as well as husbandry practices, human-animal interactions and individual animal agency. These checklists can be applied to most wild species and, as Ackonc-AWA, fit the prescriptive model (20) since they are helpful in the development of action plans to improve welfare conditions and to set priorities. However, one of the main challenges of working with wildlife is the great diversity of species, with characteristics and needs that are very different from one another (38). Therefore, similar to the work of Asher et al. (27), Clegg et al. (28), Salas et al. (22), Yon et al. (29), and Padalino and Menchetti (30), Ackonc-AWA provides specific indications and descriptions to assist sentinels in the assessment of each indicator. The distinctive feature of Ackonc-AWA is that, notwithstanding its multi-species applicability, it proposes a standardized and detailed guide on the method to adapt, assess and rate each indicator as required by each species.

Therefore, in order to successfully implement the current protocol, prior preparation is a key stage when used on a species for the first time. This includes reviewing the most updated guidelines for the adequate maintenance of the species in captivity and its dietary, health, environmental, behavioral, and affective needs (3, 38). Sometimes this information may not be available, and it becomes necessary to search for information on the species natural history, biology, ecology, diet, sensory systems, natural habitat, social structure, ethogram, activity patterns, and most common health problems and signs of illness, considering the different life stages (25). Some preparation is also needed when assessing an individual for the first time, such as information on enclosure size and design, schematic segmentation of the enclosure according to the biological relevance of each sector, major life history events and medical records. Thus, before applying the Ackonc-AWA protocol for assessing the welfare of an individual, the sentinels should do a crucial (but guided) previous step: to adapt the protocol to the specific welfare-related characteristics and requirements of the target species. By completing a spreadsheet with the optimal conditions for the welfare of the specific species to be evaluated and by adapting the indicators included in the protocol, sentinels would be able to compare them with those observed for the assessed individual and identify potential welfare concerns or needs of improvement.

The need for prior search for information on the species and, if not available, the realization of an ethogram, could take considerable time. This time may be longer or shorter depending on the species, since for some there are husbandry manuals and abundant bibliography, and for others information is very scarce or absent. This prior preparation could be seen as a limitation in comparison to other tools. However, it is important to note that this procedure is done only once at the beginning of the assessment and then the sentinels use the protocol adapted to the species of interest, without the need to go back to the literature for each assessment. In the field trials, the average time used by the sentinels was 51.32 min +/– 29.36 min for the initial form and 58.32 min +/– 23.11 min for the follow-up form. Even with the addition of activity budgets (60 min in total), an increase in the time required for assessment is not expected, as many indicators can be assessed during the same observation. Nevertheless, this should be evaluated in further studies.

Ackonc-AWA implementation cost is low, it is non-invasive/intrusive and takes relatively little time. Although these are all desirable qualities for any animal welfare assessment protocol (39), they could become an essential prerequisite for a welfare evaluation tool intended to be applicable on a daily or weekly basis in institutions with such dissimilar realities, in terms of financial and human resources, as those found in Latin American zoos.

It should be noted that the Ackonc-AWA protocol includes some indicators that can be assessed by close observation and even palpation (i.e., body condition score). This is so that future users of the protocol are able to collect the information in the most practical way for them, as many ABM can be assessed by training and conditioning or during a scheduled veterinary capture. Given that zoos frequently train animals to cooperate in veterinary maneuvers without the need for physical or chemical restraint, and that there is abundant scientific evidence indicating that operant conditioning training is another strategy to improve animal welfare in zoos and the human-animal bond, and is even a form of environmental enrichment (40–42), close observation and hands-on assessment are not discarded. However, the protocol has been specifically designed so that contact with the animal is not essential to perform the welfare assessment, and was tested hands-off. This flexibility reinforces the practicality and non-invasiveness attributes of the protocol.

In order to further increase its practicality, Ackonc-AWA was designed in two forms: initial and follow-up. Although, as discussed above, preparation requires some time, once the protocol has been adjusted to the species under study, the follow-up form can be applied as often as necessary, even on a daily basis. Its practicality and low cost of implementation is partly based on the fact that, subject to prior training, it can be applied by the institution's own personnel and done in the context of their daily duties.

In this regard, a core component of developing and using animal welfare assessment tools in zoos is to leverage the experience and expertise of the staff (13, 43, 44). Zoos often have keepers with years of experience working with a particular species, as well as the opportunity to observe individuals over long periods of time and in a variety of contexts. As such, they usually develop skills and abilities to detect and integrate subtle changes in behavior, posture, attitude, expression, or movement (13). In addition, many of the indicators to be assessed are part of their daily tasks, so zookeepers do not need to coordinate with another member of zoo staff the proper moment to do it (e.g., to assess response to environmental enrichment). Furthermore, the inter-observer agreement of ratings performed by zookeepers on zoo animals has been examined and high levels of agreement have been reported (45–48). Therefore, the Ackonc-AWA protocol was conceived to benefit from a systematic collection of information by experienced zookeepers.

Simplicity of implementation is also a key factor for the feasibility of animal welfare assessment protocols. Although the overall feasibility results were positive, adequate training and coaching could be implemented to reduce some of the difficulties encountered by sentinels when filling out the forms in animal welfare assessments. As demonstrated by Rodríguez Ruiz and Heredia Rico (49), training increases reliability of the results and reduces the protocol application time, which becomes relevant since the accuracy of the measurement decreases as the observer gets tired (50). In this study, although inexperienced sentinels received a short training (4 h), the average difficulty values for both forms were relatively low, suggesting that they could be further improved with longer training. This could be explored in future studies.

The indicators most frequently reported as difficult to assess were “Behavioral diversity”, “Defecation behavior” and “Micturition behavior”. In order to simplify the assessment of “Behavioral diversity”, the use of activity budget sheets through focal (individuals) and scan (group) sampling was incorporated, as it is an objective, quantitative and validated method for animal welfare assessment in zoos (37, 51, 52), as well as for “Space use” (53, 54). Regarding “Defecation behavior” and “Micturition behavior”, the difficulty could reflect their relatively low frequency of occurrence during brief observation periods. However, we consider that they are indicators of great value for the welfare assessment of animals and their inclusion was deemed necessary. Abnormalities in these two behaviors could be related to somatic conditions and pain or distress, arousal and fear (55). In addition, Ackonc-AWA was intended to be applied by zookeepers, who routinely have the opportunity and the skills to detect these subtle changes in the behavior of the animals in their care (13), which would overcome this constraint. Although the addition of the activity budget sheets could potentially increase the total time required for the assessment, it provides greater robustness in assessing the aforementioned indicators as well as greater flexibility to use the protocol on crepuscular and nocturnal species, through direct or recorded observations. Moreover, the proposed behavioral budget form was designed to reduce time consumption and to be applied in institutions with time constraints, since its interpretation is left to a trained person (analyst) other than the sentinels.

The need for an analyst can also be discussed as a possible disadvantage. Nevertheless, the analysis and interpretation of the information obtained from behavioral budgets has been widely used in zoos, and many of the institutions in Latin America have highly trained personnel within their staff to perform this task.

To assess affective states, Ackonc-AWA proposes a joint approach of the behavioral domain with the mental domain. This is because some affective states are directly or indirectly assessed in this protocol using behavioral indicators. Due to the type of institutions for which this protocol was designed, the importance of assessing affective states in relation to the human-animal bond is emphasized. The effects of the visitors and zookeepers over the animals' experiences and their consequent welfare state are addressed in the protocol through two ABM indicators: Reaction to strangers and Interaction with zookeepers. As stated by Mellor et al. (31), Domain 4 (Behavioral Interactions) is intended to capture behavioral outputs as indices of animals' perceptions of their external circumstances. Hence, the inclusion of Reaction to strangers aimed at evaluating the affective experiences that animals may have when they direct their attention toward unfamiliar people. This could be recognized as behaviors associated with negative states (i.e., freezing, hypervigilance, fear, hiding, and aggressiveness). Behaviors associated with positive states could also be found, as animals actively seek interaction with such strangers. Regarding the indicator Interaction with zookeepers, it is relevant to assess how the animals respond to the staff with whom they are familiar: whether they respond to calls, remain indifferent or display behaviors associated with negative affective states such as those mentioned above.

All of these responses tend to offer an approach to affective states in relation with the interactions that animals and humans have. In the future, further interventions on negative or positive human attributes and attitudes toward animals could be useful to address this issue from another perspective, in order to acquire a MBM that could operate as a welfare predictor.

The qualitative nature of this protocol may be considered controversial. Observer ratings are scores given to a variable using units of measurement defined by the researchers. Since they involve subjective judgments, some researchers question whether they can be trusted to reflect reality in an unbiased manner (56). However, several studies have shown that observer ratings can be reliable and valid [e.g., (46, 57–60)]. They have been widely employed to assess physical traits [e.g., (61, 62)], health-related variables [e.g., (63–65)], animal personality [e.g., (46, 59)], behavioral patterns [e.g., (45)], and a number of variables relevant to animal welfare [e.g., (66)]. In addition to their practicality, non-invasive nature and low cost (56), observer ratings can be used to integrate multimodal information across time and situations, and for constructs that would otherwise be very difficult to assess [e.g., pain: (65, 67)]. Furthermore, this method seems to be useful for most species that have been tested so far (56). Biases are indeed a risk, especially when the ratings could reflect the observer's or institution's own care of the animals (68–70). Nevertheless, this risk can be minimized by careful wording of the questions to be answered, development of appropriate scales, selection and training of observers, and field testing (56).

With regards to the final assessment results, Ackonc-AWA provides a representation of an animal's welfare and a temporal component that is easy to read and allows tracking changes over time, making it possible to differentiate between problems that affect animal welfare at the current time and those that pose a risk to animal welfare in the medium and long term. As a protocol with a prescriptive approach, it does not give a final numerical result, but looks at each indicator in order to identify potential welfare concerns, which prevents the institution from settling for an acceptable overall result that could be deceiving and could pose a severe threat to animal welfare. For example, a zoo that scores 8 out of 10 might be satisfied with the idea that it has a good overall score and not work on establishing a plan to improve those indicators that were found to be compromised. The situation of these compromised indicators could become chronic and begin to impact negatively on others that were adequate. On the other hand, by letter-marking indicators it is easy to identify those that require immediate resolution and establish a prioritization plan. The proposed 3-point scale score would facilitate a fast and practical prioritization of the identified welfare concerns, and to tag the more urgent correction actions.

The implementation of Ackonc-AWA in zoos could be very useful for decision-making within the ethical frameworks of compassionate conservation and conservation welfare, by evaluating the impact of different actions and situations, and guiding future decisions, so to ensure that *ex situ* conservation efforts do not harm (or do as little as possible) the welfare of individuals (8, 71).

## Conclusion

This study aimed to develop, test in the field, and describe an animal welfare assessment protocol for wild animals under human care, that can be applied on a daily basis, noninvasively, and at a low cost, under the prescriptive model. Therefore, a protocol structured in two forms (one exhaustive and other for routine use) was tested on 14 species of different taxa housed in a zoo in Argentina. Representatives from different areas of the institution as well as 3 of the authors participated in the test. It was possible to demonstrate the feasibility and test-retest reliability of the protocol. However, due to time limitations of the institution staff, its inter-observer reliability has yet to be tested.

As a result of this process, Ackonc-AWA, a multidimensional protocol for welfare assessment in multiple animal species under human care, was obtained. This proposal offers an intermediate solution between protocols that are easy to apply yet rely entirely on the judgment of the assessors, and validated but species-specific protocols that are useful only for assessing the species for which they were developed.

Further applications of the described welfare assessment tool in other species and different institutional contexts will reinforce the validation of the proposed measurements and allow the systematic and routine evaluation of animal welfare in zoos.

## Data availability statement

The original contributions presented in the study are included in the article/Supplementary material, further inquiries can be directed to the corresponding author/s.

## Ethics statement

Ethical review of the project was requested to the Institutional Committee for the Care and Use of Laboratory Animals (CICUAL) of the Faculty of Veterinary Sciences of the University of Buenos Aires, and a review exemption was granted given the observational nature of the project. The study focused on the non-invasive/intrusive assessment of animal welfare, so no interventions of any kind were carried out on the animals. There were no potential adverse effects, nor foreseeable risks or hazards associated with this project, with regards to animal, plant and/or human wellbeing. The participation of zoo staff in this study was completely voluntary and written informed consent was obtained from all participants. The survey responses were strictly confidential and data from this research was reported only in the aggregate. The information was coded and remains confidential.

## Author contributions

DR, AF, and LR contributed to the elaboration of the first drafts of the protocol, interviewed the zoo staff, met with the zoo staff/focus group to test validity, designed the on-field test, trained the staff as sentinels, organized the scheduled observations for all the sentinels, did field work as sentinels, analyzed the results of the observations including feasibility results, and elaborated the Ackonc-AWA protocol. DR contacted and coordinated actions with the institution where the pilot test was conducted and wrote and edited the first draft of this manuscript. LR and AF wrote sections of the manuscript and edited the first draft and manuscript. CB was responsible for the statistical analysis of the results for test-retest reliability and edited the manuscript. OT-P collaborated as the main counselor for methodological aspects of the study and edited the manuscript. All authors contributed to manuscript revision, and read and approved the submitted version.
